# Characteristics of Microbial Factors of Healthcare-Associated Infections Including Multidrug-Resistant Pathogens and Antibiotic Consumption at the University Intensive Care Unit in Poland in the Years 2011–2018

**DOI:** 10.3390/ijerph17196943

**Published:** 2020-09-23

**Authors:** Agnieszka Litwin, Olga Fedorowicz, Wieslawa Duszynska

**Affiliations:** 1 Microbiology Laboratory, University Hospital Wroclaw, 50-556 Wroclaw, Poland; agalee9@op.pl; 2 Department of Clinical Pharmacology, Wroclaw Medical University, 50-367 Wroclaw, Poland; olga.fedorowicz@umed.wroc.pl; 3 Department and Clinic of Anesthesiology and Intensive Therapy, Wroclaw Medical University, 50-367 Wroclaw, Poland

**Keywords:** multidrug-resistant pathogens, healthcare-associated infections, intensive care unit, DDD

## Abstract

**Introduction:** In recent years, an increase in healthcare-associated infections (HAIs) caused by resistant pathogens, which is a clinically troublesome trend, has been observed. The aim of the study was to analyze the microbial factors of HAIs and the drug resistance of microorganisms to selected antibiotics and their consumption. **Material and Methods:** The retrospective study included 3708 patients hospitalized in the intensive care unit (ICU) of the University Teaching Hospital in Wroclaw, who were diagnosed with 742 HAIs in the period from 1 January 2011 to 31 December 2018. The aim of the study was the analysis of microorganisms isolated in the respective clinical forms of HAIs, including the occurrence of “alert pathogens”, presence of multidrug-resistant (MDR) strains, and consumption of selected antibiotics. **Findings:** During the study period, 846 microorganisms were cultured in patients with HAIs, and among them, Acinetobacter baumannii MDR represented 31.8%; Klebsiella pneumoniae ESBLs, 11.3%; Pseudomonas aeruginosa MDR, 4.1% and MRSA, 2.2%; and *Enterococcus* spp. vancomycin-resistant enterococci (VRE), 1.3%. Among all the pathogens, Gram-negative bacteria (GNB) were dominant (71.6%). Gram-positive bacteria and fungi accounted for 21.6% and 7%, respectively. The total number of strains responsible for ventilator-associated pneumonia (VAP), urinary tract infection (UTI), and central line-associated blood stream infection (CLA-BSI) was as follows: 458 (54.1%), 274 (32.4%), and 114 (13.5%), respectively. Among the etiological factors of VAP, there was a prevalence of A. baumannii MDR (41.9%), as well as in the case of UTI (21.9%). With regards to CLA-BSI, MRCNS (29.8%) was the dominant pathogen. The “alert pathogens” accounted for 54.7% of all the analyzed strains. The MDR strains represented 72.6% and 9.7% among A. baumannii and P. aeruginosa, respectively. In the years 2011 vs. 2018, an increase in infections with MDR bacilli was observed, 34.6% vs. 61.0% (*p* = 0.0008), respectively, including A. baumannii MDR 16.54% vs. 34.56 % (*p* = 0.0009) and Enterobacterales ESBL+/AMPC 11.8% vs. 15.44 % (*p* = 0.3921). Resistance to methicillin was confirmed in 35.2% of S. aureus strains. Resistance to vancomycin was found among 30.9% of *Enterococcus* spp. The observed period was marked by an increase in the consumption of carbapenems: 197.7 vs. 235.9 defined daily dose (DDD)/1000 patients-days. **Conclusions:** Gram-negative bacteria were found to be dominant pathogens in healthcare-associated infections. The most frequently cultured pathogens were multidrug-resistant *A. baumannii, K. pneumoniae* ESBL(+), and *P. aeruginosa*. The study showed an increase in the incidence of “alert pathogens” and MDR bacilli, as well as the tendency of a growing resistance to antibiotics during the observed period. Microbiological analysis of HAIs and the consumption of antibiotics is the necessary element of the proper antibiotic policy in hospitals.

## 1. Introduction

Infections affect about 45–51% of patients treated in intensive care units (ICU) [1,2]. The etiology of healthcare-associated infections (HAIs) is traced to the invasiveness of diagnostic and therapeutic techniques and the severity of patients’ conditions. Clinical forms of HAIs, which are most frequently subjected to monitoring in ICU patients, include ventilator-associated pneumonia (VAP), catheter-associated urinary tract infections (UTI), and central line-associated blood stream infections (CLA-BSI) [3,4]. The multicenter European Prevalence of Infection in Intensive Care study—EPIC (1992) showed predominance of infections (44.8% infected patients including 20.6% with ICU-acquired infections) caused by Gram-negative bacteria (GNB) (63.1%). In the same study *Enterobacteriaceae* were the most frequent infection pathogens (34.4%), while strains of *Acinetobacter baumannii* and *Pseudomonas aeruginosa* accounted for 9% and 28.7% of infections, respectively, whereby staphylococcal infections were represented at the level of 30.1% and fungal infections comprised 17.1% [2]. Similarly, in the worldwide study The Extended Prevalence of Infection in Intensive Care—EPIC II (2007) (51% infected patients), infections with Gram-negative etiology were predominant and were found among 62% of patients; 47% of patients were infected with Gram-positive strains and 19% of patients had infections with the fungal etiology. The percentage of MRSA in the same study was 10.2%, whereas the percentage of GNB with the extended spectrum beta-lactamase resistance mechanism was not specified [2]. According to the last worldwide multicenter EPIC III study (2017) (54% infected patients including 22% with ICU-acquired infections), GNB were still the most frequently found pathogens among ICU patients (67%), whereas Gram-positive pathogens (37%) and fungi (16%) were found less frequently [5]. The results of the Polish register of cases of sepsis in the years 2003–2009 showed that GNB accounted for severe infections in 58% of patients; Gram-positive bacteria (GPB) caused infections in 34% of patients and fungi resulted in infections in 16% of patients [6]. In the last decade, “alert pathogens”, which are defined as microorganisms playing a significant role in the development of resistance to many groups of antibiotics, and which were referred to as ESKAPE in the year 2008 after the first letters of their names: *Enterococcus faecium*, *Staphylococcus aureus*, *Klebsiella pneumoniae*, *Acinetobacter baumannii*, *Pseudomonas aeruginosa*, and the *Enterobacterales* family, are a serious clinical problem worldwide [7].

In certain regions of southern and central Europe, in patents treated in ICUs, we can observe an increase in the resistance related to strains of GNB, among which *A. baumannii* MDR was prevalent [8,9,10]. Moreover, an increase in infections caused by *K. pneumoniae* ESBL(+) and vancomycin-resistant enterococci (VRE) as well as MDR non-fermenting bacilli (*Pseudomonas* spp., *Acinetobacter* spp.) is a troublesome trend [8,11]. According to published data, in the last decade, the continuous pre-dominance of HAIs caused by strains of GNB (*A. baumannii, P. aeruginosa, K. pneumoniae*) was observed, among which the percentage of MDR bacteria was very high (in Serbia, 88.9–97%; Egypt, 42.5–92.8%; Greece, 92.9%; India, 35–68%; Poland, 98.36% (*Acinetobacter* only), Italy, 66.7%; the U.S., 14.6–73.1%) [10,11,12,13,14,15,16]. During the same period, the frequency of HAIs with MRSA etiology was variable and amounted to 2.7% in Greece, 4.56% in Egypt, 10% in Poland, 13.8% in France, 25.8% in Spain, and 10.3% in Germany [12,13,17,18,19]. Microbiological analysis of infections (also called “microbiological mapping”), which is the subject of this paper, aims to evaluate the epidemiological situation in the hospital unit and is a necessary element of the proper antibiotic policy [20]. Despite recurrently published reports on microbial resistance by European Center Disease Control as well as Center Disease Control, the data on continuous microbiological analysis of infections from University Hospitals from Europe are lacking.

The aim of the presented study was to analyze the microbial factors of HAIs recorded during 8 years of observation at the ICU, their drug resistance to selected antibiotics, and the consumption of selected antibiotics expressed in the defined daily dose (DDD). It is important to become familiar with trends related to the occurrence of alert pathogens and their sensitivity, to apply remedies aimed to prevent the spread of resistant strains, and to establish procedures for empirical antibiotic therapy [21].

## 2. Material and Methods

### 2.1. Data Collection

The retrospective study covered 3708 patients (1464 females and 2244 males; average age 60.8 ± 18 years) who were hospitalized at the intensive care unit of the University Teaching Hospital in Wroclaw, and who were diagnosed with HAIs in the period between 1 January 2011 and 31 December 2018. The database was drawn up on the basis of the results of microbiological tests performed at the Microbiological Laboratory of the University Teaching Hospital and the electronic records of the hospital. The study also uses the data regarding infections collected during the registration of HAIs in the form of monthly unit reports. The retrospective study was prepared with the consent of the Bioethical Committee of the Medical University in Wroclaw no. KB-576/2016. The Institutional Ethics Committee’s consent included approval for publication of the data. The patients’ written consents were not required by the Ethics Committee of Wroclaw Medical University because data collection was a part of the infection control program and a statement covering patients’ data confidentiality was fully respected during data collection and the preparation of the manuscript. 

In order to diagnose the infection or colonization, on admission to the ICU and then once a week, we subjected each patient to the sampling of materials for routine microbiological tests (throat swab, bronchial secretion, urine, blood, and rectal swab) and, additionally, depending on the suspected clinical form of the infection: blood, venous catheter tip, swab from a wound, fluids from body cavities, or cerebrospinal fluid. The study analyzed pathogens responsible for infections only, not for colonization. The following HAIs have been subjected to microbiological analysis in this paper: VAP, UTI, and CLA-BSI.

### 2.2. Microbiological Diagnosis of Infections 

VAP was diagnosed microbiologically using mini-bronchoalveolar lavage (mini-BAL) or BAL with >10^4^ colony forming units (CFU)/mL. UTI was diagnosed in cases of bacterial loads in urine >10^3^ or <10^5^ CFU/mL, with the presence of no more than two pathogens and pyuria. CLA-BSI was diagnosed in cases of a positive blood culture and >15 CFU in a semi-quantitative method or >10^3^ CFU/mL in a quantitative method from the tip of the vascular catheter [22]. Microbiological diagnostics of infections was carried out by a certified microbiological laboratory of the University Hospital in Wroclaw, according to recommendations of the European Committee on Antimicrobial Susceptibility Testing (EUCAST) for methodology of diagnostic methods and interpretation [23]. Identification of Gram-positive and Gram-negative bacteria was carried out with the use of specific/appropriate biochemical tests and by applying automatic method (Gram-negative and Gram-positive card) in the Vitek 2 automatic system, according to Good Laboratory Practice principles. Susceptibility of microorganisms was also determined manually with the use of disk diffusion method on Muller–Hinton (BioRad, Berkley, CA, USA) substrate and automatic method (AST-N332 card) in the Vitek 2 system.

### 2.3. Data Analysis 

“Alert pathogens” were defined as very difficult in terms of treating bacterial strains, including *A. baumannii, P. aeruginosa, K. pneumoniae, Enterobacter* spp., MRSA, and *Enterococcus faecium* according to the previously published definition [7]. MDR of the *A. baumannii* and *P. aeruginosa* strain was defined as resistance of the strain to at least three groups of antibiotics [9].

Strains of *A. baumannii* MDR, *P. aeruginosa* MDR, and *K. pneumoniae* ESBL(+) were analyzed in terms of the changing resistance (in the years 2011–2018) to carbapenems (imipenem, meropenem), quinolones (ciprofloxacin), and aminoglycosides (amikacin, gentamycin). The paper also presents the assessment of consumption of selected antibiotics (amikacin, ciprofloxacin, imipenem, meropenem) in the years 2011, 2014, and 2018, calculated as the defined daily dose per 1000 patients-days. 

The data were statistically analyzed using Statistica 10.0 (StatSoft. Inc. Tulsa, OK, USA). Variables were analyzed using Pearson’s chi-square test or the chi-square test with Yates correction as appropriate. *p* < 0.05 was considered significant. 

### 2.4. Ethical Approval

Approval of this study (no. KB-576/2016) was given by the Bioethics Committee of the Wroclaw Medical University (Poland). The study was based on anonymous data, including laboratory results that were used retrospectively. The Bioethics Committee of Wroclaw Medical University consent included approval for publication of the data without need of statement or consent from participants.

## 3. Findings

### 3.1. The Most Prevalent Microorganisms Responsible for HAIs

In total, 3708 patients were hospitalized at the ICU during the analyzed period. Healthcare-associated infections were diagnosed in 20% patients. In this study, 742 HAIs caused by 846 microorganisms were detected. In 89 patients, more than one infection was observed, and 104 infections were caused by more than one pathogen. The total number of strains responsible for VAP, UTI, and CLA-BSI amounted to 458 (54.1%), 274 (32.4%), and 114 (13.5%), respectively. The most frequently isolated groups of microorganisms, irrespective of the clinical form of the infection, were GNB (71.4%), Gram-positive cocci (21.6%), and fungi (7%). Among all the microorganisms detected, *Acinetobacter baumannii* MDR consisted of 31.8%, *Klebsiella pneumoniae* ESBLs of 11.03%, *Pseudomonas aeruginosa* MDR of 4.1% and MRSA 2.2%, and *Enterococcus* spp. VRE of 1.3%. 

Among the etiological factors of VAP, Gram-negative, non-fermenting *A. baumannii* MDR (41.9%) prevailed, followed closely by *P*. *aeruginosa* (13.7%) and *K. pneumoniae* ESBL(+) (11.6%). In the case of UTI, MDR *A. baumannii* (21.9%) and *Candida* spp. (13.1%) were isolated most frequently. In CLA-BSI, methicillin-resistant, coagulase-negative staphylococci (MRCNS) (29.8%) and *K. pneumoniae* ESBL(+) (13.2%) were dominant (Table 1).

### 3.2. Changes in the Prevalence of “Alert Pathogens” between 2011 and 2018

Among 846 strains resulting in HAIs, the “alert pathogens” comprised 54.7% (463). A significant increase in their number from 34.6% in 2011 to 61.0% in 2018 was observed (*p* = 0.0008). During the total study period, the number of infections caused by *A. baumannii* MDR (*p* = 0.0009), MRSA (*p* < 0.0001), and VRE (*p* < 0.0001) significantly increased (Table 2).

In the year 2011, the most frequently identified “alert pathogens” included *A. baumannii* MDR (44.7%), and then *Enterobacterales* ESBL(+) (34%) and *P. aeruginosa* MDR (18.2%). 

**Figure 1:**
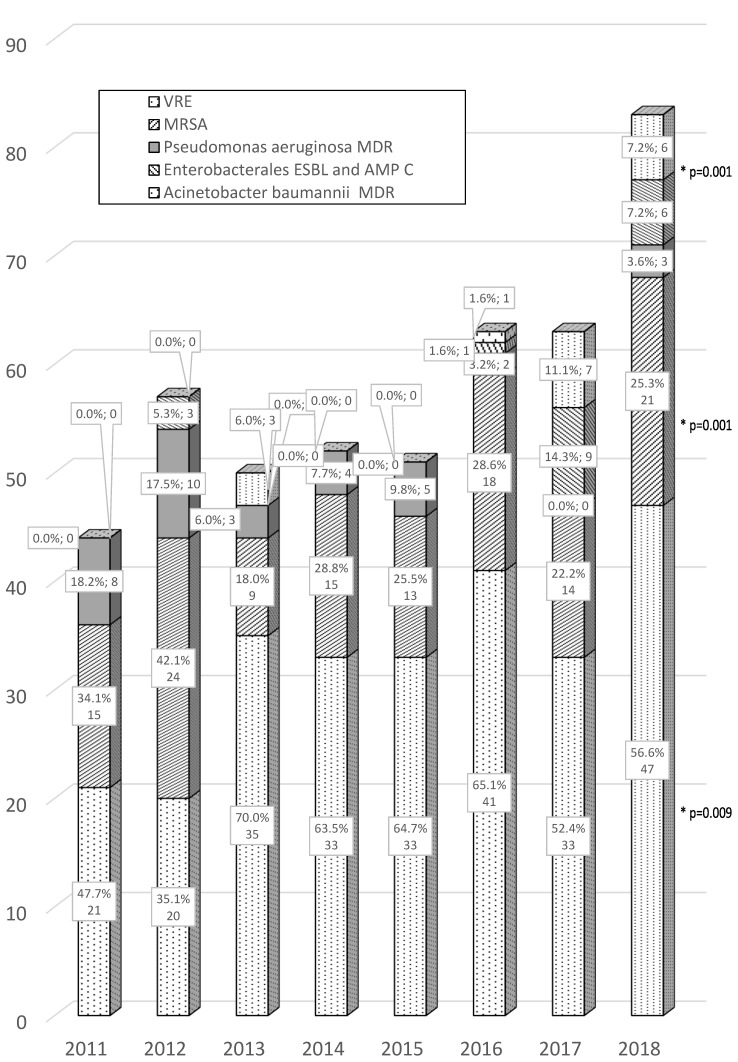
Alert pathogens isolated from hospital-acquired infections in the years 2011–2018. The data were presented as numerical values and percentage values of the total number of multidrug-resistant (MDR) strains in subsequent years. Legend: * *p* was calculated for comparison of the year 2011 vs. 2018. Only significant increase was marked.

Analysis of the last two years of the study (2017/2018) demonstrated the continuous predominance of *A. baumannii* MDR among the cultured “alert pathogens”, representing 52.4%/56.6%, respectively, and *Enterobacterales* ESBL(+) representing 22.2%/25.3%. During the same period, there was also an observed increase in the presence of *Staphylococcus aureus* (MRSA) 14.3%/7.2% and *Enterococcus* spp. VRE 11.1%/7.2% among “alert pathogens” (Figure 1). 

### 3.3. ”Alert Pathogens” Antibiogram

Out of 263 studied *A. baumannii* strains, as many as 74.9% showed resistance to carbapenems (the value for imipenem and meropenem was averaged), with 97% showing insensitivity to fluoroquinolones and 96.6% demonstrating resistance to aminoglycosides (the value for gentamicin and amikacin was averaged). In the analyzed period, an increase in resistance *A. baumannii* to all the three analyzed groups of antibiotics was observed (10-fold increase in resistance to meropenem, more than twofold to ciprofloxacin and more than threefold to amikacin). The second pathogen most frequently found was *K. pneumoniae* ESBL(+), which demonstrated 93.7% resistance to fluoroquinolones and insensitivity to aminoglycosides only at the level of 41.7%. Among the strains of *K. pneumoniae* ESBL(+), we observed no resistance to carbapenems. In the analyzed period, a slight decrease in resistance of *K. pneumoniae* to amikacin was observed. On the other hand, *P. aeruginosa* MDR (the third most frequently cultured infectious pathogen) demonstrated the highest resistance to carbapenems (94.3%), with insensitivity to aminoglycosides in 71.4% and to fluoroquinolones in 85.7% of the cultured strains. The analysis of resistance *P. aeruginosa* showed a slow decline in resistance to meropenem and ciprofloxacin in the observed period (Table 3).

Analysis of MRSA strains (19/4.1%) demonstrated 100% sensitivity to vancomycin. *Enterococcus* spp. VRE, which occupies the last position among the analyzed alert strains, demonstrated 100% sensitivity to linezolid, with simultaneous 100% resistance to ampicillin.

Overall, the percentages of MDR bacteria among all isolated strains of *A. baumannii* and *P. aeruginosa* were 72.6% and 9.7%, respectively. *Enterobacterales* ESBL(+) represented 21.4% of all GNB. During the observed period, an significant increase in incidence of infections of *A. baumannii* MDR( 21/127) 16.54% vs. (47/136) 34.56% (*p* = 0.0009) and non-significant *Enterobacterales* ESBL+/AMPC (15/127) 11.8% vs. (21/136) 15.44% (*p* = 0.3921) was observed in the years of 2011 vs. 2018. Overall, during the aforementioned period, the number of infections caused by MDR bacilli increased significantly from 34.6% to 61.0% (*p* = 0.0008). Resistance to methicillin was confirmed in 35.2% of *S. aureus.* Among MRSA, no resistance to vancomycin was observed, while at the same time it was found among 30.9% of *Enterococcus* spp.

### 3.4. Selected Consumption of Antibiotics among ICU Patients

The assessment of the consumption of selected groups of antibiotics converted to DDD/1000 patients-days in the years 2011–2018 is presented in Table 4. Analysis demonstrated a significant increase in the consumption of carbapenems (imipenem/meropenem) 197.7 vs. 235.9 DDD/1000 patients-days (*p* = 0.039), a fourfold reduction in the consumption of ciprofloxacin (*p* < 0.0001), and a slight decrease in the consumption of amikacin. 

## 4. Discussion

Healthcare-associated infections are a problematic issue for all hospitals in Poland, as well as in hospitals all over the world. Because of the severity of patients’ conditions, ICUs deserve particular attention. Not only is the monitoring of HAIs and microbial factors of healthcare-associated infections (with particular consideration for “alert pathogens”) of cognitive and clinical significance, but it is also an accreditation requirement for hospitals and a recommendation of the European Parliament [24].

The first parameter analyzed in the paper was the observation that the most frequently isolated groups of pathogens, irrespective of the clinical form of the infection, included GNB (71.4%), Gram-positive cocci, and fungi. While a definite predominance of GNB was found in VAP and UTI, in the case of CLA-BSI infections, the analysis demonstrated a comparable number of GNB and GPB. The results of our studies do not differ significantly from other published studies, which show that the dominant HAIs include pneumonias followed by UTI and CLA-BSI [3,25,26]. With regards to the domination of GNB in the pathogenesis of HAIs, the results of our study correspond with the results of the Polish multi-center study (PPIC), where the GNB were responsible for 64.1% of infections, while Gram-positive bacteria accounted for 31.8% of infections, with the percentage of infections caused by fungi being 4.1% [18]. They also correspond with the results of another Polish multi-center study, where GNB accounted for 69.2% of infections and Gram-positive bacteria accounted for 26.8% [27]. The results of international registers of HAIs do not present the aforementioned division, however, high percentages of GNB in the pathogenesis of VAP and UTI demonstrate the predominance of these pathogens in the pathogenesis of HAIs all over the world [3,25,26].

The second variable analyzed in the paper was related to the determination of microbial factors responsible for various clinical forms of HAIs. It was proven that the dominant etiological factor of VAP was *A. baumannii* MDR (41.9%), *K. pneumoniae* ESBL (+), and *S. aureus* (10.3)%, which corresponds with the results of another study from a Polish center, where the percentage of *A. baumannni* was 41%, *P. aeruginosa* was 12%, and *S. aureus* was 9% [28]. The data published by ECDC indicate that the problem of a large number of lung infections with *A. baumannii* etiology refers to Poland (35.7%), while the summed up microbiological data indicated that in Europe, *P. aeruginosa* (20.8%), *S. aureus* (17.8%), and *K. pneumoniae* (16.1%) are dominant in the pathogenesis of VAP, and *A. baumanii* is only 4.1% [26]. Analysis of etiological factors of UTI in our study clearly showed that infections caused by *A. baumannii* MDR (21.9%) and then *Candida* spp., *K. pneumoniae* ESBL(+), and VRE were the most serious problem. The ECDC report indicates that *Enterococcus* spp. was the most frequent pathogen, which caused UTI (26.3%) in Poland in the year 2016 [26]; however, this was not confirmed by our study. The results of our study in this respect differ from the results of the ECDC report from the year 2017, in which *Escherichia coli* (32.1%), *Enterococcus* spp. (20.6%), and *Klebsiella* spp. (14.5%) were predominant, while infections with *Candida* spp. and *A. baumannii* etiology were observed in 2.8% and 1.4% of cases, respectively [29].

In the analysis of pathogens responsible for CLA-BSI in our study, MRCNS were definitely predominant (29.8%), followed by *K. pneumoniae* ESBL(+) and *A. baumannii* MDR. This correlates with 10-year studies conducted in a hospital in the southern part of Poland, where the percentage of MRCNS responsible for CLA-BSI was the highest (44.2%), as well as with the data regarding this type of infection in ICUs in Poland, where the GPB are the causes of 43% infections, of which 58% are caused by MRCNS [28]. Similarly, the ECDC report from the year 2017 indicates that the most frequent pathogen of CLA-BSI in Europe is MRCNS (23.6%), then *Enterococcus* spp. (14.9%), *Klebsiella* spp. (12.4%), and *S. aureus* 12%, while *A. baumannii* represents only 2.3% of the pathogens [29].

The third issue studied in this paper was the resistance of alert strains to antibiotics.

The problematic fact is that a significant increase in the resistance of non-fermenting rods to carbapenems has been observed both in Poland and globally [25,30]. This phenomenon has also been observed in our own studies. The comparative analysis of the data from reports on the monitoring of drug sensitivity of pathogens isolated from invasive infections indicates that in Poland in 2014, the percentage of strains resistant to carbapenems among *A. baumannii* was 53.4%, and *P. aeruginosa* 27.6%, and in the year 2017, the resistance was 67.4% and 24.2% for *A. baumannii* and *P. aeruginosa*, respectively [30]. During the analyzed period, the resistance of the strains of *A. baumannii* (94.3%) and *P. aeruginosa* (74.9%) to carbapenems was higher than in Europe in the EARS-Net study (33.4% and 17.4%, respectively), similar to the resistance of these strains to fluoroquinolones and aminoglycosides [30].

The present study demonstrated that the HAIs causing *K. pneumoniae* ESBL(+) require carbapenem therapy as the basic option, as no resistance to these antibiotics has been proven. Another noteworthy phenomenon is the high resistance of *K. pneumoniae* to fluoroquinolones (93.7%), which unambiguously excludes these antibiotics from the empirical therapy. On the basis of the EARS-Net base, resistance to carbapenems among the strains of *K. pneumoniae* in Polish hospitals was found to be 6.4%, while in low-income countries (INICC report), as well as in Italy and Greece, it is much higher, at 19.6%, 29.7%, and 64.7%, respectively [29,31]. In the EARS-Net study from the year 2017, the lowest resistance of *K. pneumonaiae* to fluoroquinolones (66.3%) and aminoglycosides (55.5%) was observed in Europe, whereas in our study this resistance was slightly lower (41.7%) [30]. The percentage of MDR bacteria among the strains of *A. baumannii* in our study (72.6%) was higher than in the EARS-Net study in Europe (43.2%). On the contrary, the percentage of *P. aeruginosa* MDR in our study (9.7%) was lower than in Europe (13.3%) and in Poland (22.8%) [30]. The MRSA infections in our study were observed most frequently in patients with VAP (6.3%/3.9%), while *Enterococcus* spp. VRE was cultured most frequently in patients with UTI (5.8%). The published study from a university center in the USA indicates that the percentage of MRSA in patients with VAP is higher (28.8%) [32]. A similarly higher percentage of infections with *S. aureus* was observed in Europe in the case of VAP (18.5%) and in the case of CLA-BSI (12.0%) [30]. Resistance of *S. aureus* strains to methicillin in our study (35.2%) was lower than in the case of CLA-BSI in the INICC report (64.7%) and CDC/NSHN report (50.7%), and higher than in the ECDC study (24%) and a study conducted by another Polish center (30.3%) [11,28,29,31]. According to the EARS-Net base, the percentage of MRSA in Europe was 16.9%, while in Poland it was 15.2%; Spain, 25.8%; Italy, 33.9%; Greece, 38.4%; and Romania, 44.4% [30]. Resistance to vancomycin in our study was found among 30.9% of strains of *Enterococcus* spp., while in the INICC register, it was observed in 18.5% of strains. In the CDC/NSHN report, this resistance was observed in 9.8% of strains, and in the CDC/NHSN microbiological report covering the years 2011–2014, it was estimated at 58.4% [3,11,31].

The final analyzed element of this paper was the assessment of the consumption of selected antibiotics on the basis of the DDD/1000 patients-days. In comparison with the year 2011, there was a fourfold decrease in the consumption of ciprofloxacin, as well as a slight decrease in the consumption of amikacin. The fact that an increase in the consumption of carbapenems during the analyzed period may correspond with an increase in alert pathogens cultured from hospital-acquired infections, mainly the ESBL-type resistance mechanism, is also noteworthy. The data from the Polish and European ICUs confirm the results of our study in that carbapenems and fluoroquinolones are most frequently used in this group of patients [30,33].

Therefore, a multi-level strategy aimed at reducing the occurrence and spread of highly resistant bacteria in hospitals is essential, as stated in the Recommendation of the European Union (EU) Council on patient safety, including the prevention and control of healthcare-associated infections [34].

## 5. Conclusions

- The most frequently isolated group of microorganisms in patients with HAIs, regardless of the clinical form of the infection, was Gram-negative bacilli (71.4%).
- The total number of strains responsible for VAP was the highest, being slightly higher (54.1%) than for UTI and BSI together.
- The variation among etiological factors of different clinical forms of HAIs was proven. Among the pathogens that are responsible for VAP, Gram-negative MDR *A. baumannii* and *K. pneumoniae* ESBL(+) were dominant. In the case of UTI, *A. baumannii* MDR and *Candida* spp. were most common, whereas in CLA-BSI, MRCNS and *K. pneumoniae* were most frequently isolated.
- Alert pathogens were found in about half of patients with HAIs, whereas MDR GNB were found in one-third and two-thirds of patients in 2011 and 2018, respectively.
- The analysis demonstrated an almost twofold increase in the isolation of “alert pathogens” from HAIs during the observed period
- A 10-fold increase in resistance of *A. baumannii* and a 100% sensitivity of *K. pneumoniae* to carbapenems was found.
- An increase in the consumption of antibiotics belonging to the group of carbapenems as well as a slight decrease in the consumption of aminoglycosides and the fourfold decrease in the consumption of fluoroquinolones were proven.
- The data regarding the microbiological profile of the unit and percentage of resistance allow for a more effective selection of the optimal therapy and the development of the antibiotic policy to avoid further increase in resistance among microorganisms from the hospital environment.
- The strengthening of microbiological diagnostics and monitoring of antibiotic consumption facilitates the establishment of the antibiotic policy and the development of procedures to protect against the spread of resistance mechanisms transmitted by microorganisms and, consequently, the increase in infections caused by alert pathogens.

## Figures and Tables

**Table 1:** Etiological factors of hospital-acquired infections and their location in the years 2011–2018. The data were presented as the number of isolated strains and percentage of the overall number of isolated strains.

Pathogen	VAP	UTI	CLA-BSI	Sum
	*n* (%)	*n* (%)	*n* (%)	*n* (%)
*Acinetobacter baumannii* MDR	192 (41.9)	60 (21.9)	11 (9.6)	263 (31.08)
*Pseudomonas aeruginosa*	41 (8.9)	19 (6.9)	4 (3.5)	64 (7.6)
*Pseudomonas aeruginosa* MDR	22 (4.8)	10 (3.6)	3 (2.6)	35 (4.1)
*Stenotrophomonas maltophilia*	15 (3.3)	1 (0.4)	2 (0.7)	18 (2.1)
*Klebsiella pneumoniae* ESBLs	53 (11.6)	28 (10.2)	15 (13.2)	96 (11.3)
*Enterobacter cloacae* ESBLs	6 (1.3)	3 (1.09)	3 (1.09)	12 (1.4)
*Enterobacter aerogenes* ESBLs	-	-	1 (0.4)	1 (0.11)
*Escherichia coli* ESBLs	2 (0.44)	7 (2.5)	-	9 (1.06)
*Citrobacter freundii* ESBLs	1 (0.2)	-	-	1 (0.11)
*Serratia ficaria* ESBLs	-	1 (0.4)	-	1 (0.11)
*Proteus mirabilis* ESBLs	-	3 (1.09)	-	3 (0.35)
*Proteus vulgaris* ESBLs	-	1 (0.4)	-	1 (0.11)
*Enterobacter cloacae* AMP C	2 (0.44)	-	-	2 (0.2)
*Proteus mirabilis* AMP C	1 (0.2)	1 (0.4)	-	2 (0.2)
*Proteus vulgaris*		1 (0.4)	-	1 (0.11)
*Klebsiella pneumoniae*	20 (4.4)	6 (2.2)	4 (3.5)	30 (3.5)
*Klebsiella oxytoca*	1 (0.2)	1 (0.4)	1 (0.4)	3 (0.35)
*Escherichia coli*	10 (2.2)	11 (4.0)	1 (0.4)	22 (2.6)
*Enterobacter cloacae*	5 (1.09)	3 (1.09)	1 (0.4)	9 (1.06)
*Enterobacter aerogenes*	-	-	1 (0.4)	1 (0.11)
*Morganella morganii*	2 (0.44)	-	-	2 (0.2)
*Serratia marcescens*	5 (1.09)	-	4 (3.5)	9 (1.06)
*Proteus vulgaris*	1 (0.2)	1 (0.4)	-	2 (0.2)
*Proteus mirabilis*	1 (0.2)	12 (4.4)	-	13 (1.5)
*Proteus pennerii*	1 (0.2)	-	-	1 (0.11)
*Enterobacter aerogenes*	3 (0.6)	-	-	3 (0.35)
*Candida* spp.	10 (2.2)	36 (13.1)	13 (11.4)	59 (7.0)
MSCNS	2 (0.44)	1 (0.4)	1 (0.4)	4 (0.5)
MRCNS	-	1 (0.4)	34 (29.8)	35 (4.1)
MRSA	18 (3.9)	-	1 (0.4)	19 (2.2)
MSSA	29 (6.3)	-	1 (0.4)	30 (3.5)
*Enterococcus faecalis*	2 (0.44)	13 (4.7)	5 (4.4)	20 (2.4)
*Enterococcus faecium*	9 (2.0)	7 (2.5)	-	16 (1.9)
*Enterococcus faecium* VRE	1 (0.2)	5 (1.8)	-	6 (0.7)
*Enterococcus faecium* HLAR VRE	-	11 (4.0)	-	11 (1.3)
*Enterococcus faecalis* HLAR	-	9 (3.3)	2 (0.7)	11 (1.3)
*Enterococcus faecium* HLAR	-	22 (8.0)	5 (4.4)	27 (3.2)
*Streptococcus pneumoniae*	3 (0.6)	-	-	3 (0.35)
*Granulicatella* spp.	-	-	1 (0.4)	1 (0.11)
Sum (%)	458 (54.1)	274 (32.4)	114 (13.5)	846

Legend: *n*—number of isolated strains; %—percentage of microorganisms in the total number of isolations over the respective period.

**Table 2:** Alert pathogens isolated from hospital-acquired infections in the years 2011—2018. The data were presented as numerical values and percentage of the overall number of isolates in individual years.

	2011 *n* = 127	2012 *n* = 115	2013 *n* = 80	2014 *n* = 81	2015 *n* = 86	2016 *n* = 100	2017 *n* = 121	2018 *n* = 136	Sum	*p*
*Acinetobacter baumannii* MDR, *n (%)*	21 (16.53)	20 (17.39)	35 (43.75)	33 (40.74)	33 (38.37)	41 (41)	33 (27.27)	47 (34.56)	263	0.0009
*Enterobacterales* ESBL/AMPC	15 (11.8)	24 (20.87)	9 (11.25)	15 (18.52)	13 (15.12)	18 (18)	14 (11.57)	21 (15.44)	129	0.3920
*Pseudomonas aeruginosa* MDR	8 (6.3)	10 (8.69)	3 (3.75)	4 (4.94)	5 (5.81)	2 (2)	0 (0)	3 (2.21)	35	0.1774
MRSA	0 (0)	3 (2.61)	0 (0)	0 (0)	0 (0)	1 (1)	9 (7.34)	6 (4.4)	19	<0.0001
VRE	0 (0)	0 (0)	3 (3.75)	0 (0)	0 (0)	1 (1)	7 (7.34)	6 (4.4)	17	<0.0001
Sum of “alert pathogens”	44	57	50	52	51	63	63	83	463	

Legend: *p* was calculated for relationships in the form of figures for the period between 2011 and 2018.

**Table 3:** Percentage of resistance of selected “alert pathogens” to three groups of antibiotics in the observed years.

	2011			2012			2013			2014			2015			2016			2017			2018		
	MEM	CIP	AN	MEM	CIP	AN	MEM	CIP	AN	MEM	CIP	AN	MEM	CIP	AN	MEM	CIP	AN	MEM	CIP	AN	MEM	CIP	AN
*Acinetobacter baumannii* MDR *n*/(%”R”)	4/(19)	19/(90)	13 (62)	11/(55)	19/(95)	13/(65)	33/(94)	34/(98)	31/(89)	33/(100)	33/(100)	28/(88)	32/(97)	33/(100)	28/(85)	38/(93)	39/(95)	36/(88)	31/(94)	31/(94)	31/(94)	47 (100)	47/(100)	43/(92)
*Pseudomonas aeruginosa* MDR *n*/(%”R”)	9/(45)	11/(55)	8/(40)	8/(44)	11/(61)	5/(28)	4/(57)	2/(19)	1/(14)	6/(46)	9/(69)	3/(23)	4/(33)	5/(42)	2/(17)	3/(60)	3/(60)	1/(20)	3/(21)	0/(0)	0/(0)	4/(40)	4/(40)	3/(30)
*Klebsiella pneumoniae ESBLs n*/(%”R”)	0/(0)	9/(93)	7/(74)	0/(0)	12/(92)	6/(40)	0/(0)	9/(100)	3/(33)	0/(0)	15/(100)	7/(50)	0/(0)	12/(92)	5/(46)	0/(0)	11/(78)	4/(25)	0/(0)	8/(86)	3/(29)	0/(0)	14/(95)	5/(34)

Legend: AN amikacin; CIP ciprofloxacin; MEM meropenem.

**Table 4:** Consumption of selected antibiotics in intensive care units (ICUs) in the years 2011–2018.

	DDD/1000 Patient-Days		
	2011	2014	2018
Amikacin	33.50	44.23	30.70
Ciprofloxacin	119.03	67.47	27.71
Carbapenems	197.74	228.97	236.88

Legend: DDD, defined daily dose—the average daily dose of a drug used in the therapy of various diseases for an adult weighing 70 kg.
